# A startling acoustic stimulus facilitates voluntary lower extremity movements and automatic postural responses in people with chronic stroke

**DOI:** 10.1007/s00415-018-8889-5

**Published:** 2018-05-14

**Authors:** Milou J. M. Coppens, Jolanda M. B. Roelofs, Nicole A. J. Donkers, Jorik Nonnekes, Alexander C. H. Geurts, Vivian Weerdesteyn

**Affiliations:** Department of Rehabilitation, Donders Institute for Brain, Cognition and Behaviour, Radboud University Medical Center, PO Box 9101, 6500 HB Nijmegen, The Netherlands

**Keywords:** Stroke, Automatic postural responses, Balance, StartReact, Startle

## Abstract

A startling acoustic stimulus (SAS) involuntary releases prepared movements at accelerated latencies, known as the StartReact effect. Previous work has demonstrated intact StartReact in paretic upper extremity movements in people after stroke, suggesting preserved motor preparation. The question remains whether motor preparation of lower extremity movements is also unaffected after stroke. Here, we investigated StartReact effects on ballistic lower extremity movements and on automatic postural responses (APRs) following perturbations to standing balance. These APRs are particularly interesting as they are critical to prevent a fall following balance perturbations, but show substantial delays and poor muscle coordination after stroke. Twelve chronic stroke patients and 12 healthy controls performed voluntary ankle dorsiflexion movements in response to a visual stimulus, and responded to backward balance perturbations evoking APRs. Twenty-five percent of all trials contained a SAS (120 dB) simultaneously with the visual stimulus or balance perturbation. As expected, in the absence of a SAS muscle and movement onset latencies at the paretic side were delayed compared to the non-paretic leg and to controls. The SAS accelerated ankle dorsiflexion onsets in both the legs of the stroke subjects and in controls. Following perturbations, the SAS accelerated bilateral APR onsets not only in controls, but for the first time, we also demonstrated this effect in people after stroke. Moreover, APR inter- and intra-limb muscle coordination was rather weak in our stroke subjects, but substantially improved when the SAS was applied. These findings show preserved movement preparation, suggesting that there is residual (subcortical) capacity for motor recovery.

## Introduction

Falling is a common medical complication in people after stroke. People in the chronic phase after stroke have 3–10 times greater fall rates compared to healthy peers [28]. Following an external balance perturbation, the ability to execute rapid postural responses is critical for preventing a fall [16]. These automatic postural responses (APRs) are delayed in people after stroke [8, 17] and also exhibit defective intra-limb coordination (i.e. poorer relative timing of muscle activations), which impairments have indeed been linked to their risk of falling [17, 19]. It may be possible to facilitate the APR recruitment through training, but this effect has only been reported in a single paper [18], which was published more than 10 years ago and has not been replicated since. For understanding the potential trainability of APRs in people after stroke, it would be helpful to gain insights into the underlying mechanisms of defective APR recruitment.

The prevailing hypothesis is that APRs are encoded in the brainstem, from where they can be released upon sensory signals evoked by balance perturbations, thus not involving transcortical loops [15]. Cortical structures are thought to exert an *indirect* influence on APRs by setting the gains of brainstem postural circuits (e.g. for shaping the response to context-specific needs) [15]. In this view, a reduced cortical ‘drive’ after supratentorial stroke may thus result in a lower excitability of postural circuits, which may in turn explain the delayed APRs. Yet, it is unclear whether stroke-related deficits in APR coordination can be reconciled with this notion.

In recent years, mounting evidence has been presented for a more prominent role of cortical structures in human balance control, including an important role in motor preparation [3]. These new insights raise the question whether defective motor preparation may contribute to the coordination deficits of APRs that have been observed in people after stroke. Such deficits may arise from stroke-related lesions of the corticoreticular tract (i.e. tract that runs from the premotor cortex and supplementary motor areas to the pontomedullary reticular formation) [29], resulting in defective ‘programming’ of APRs at the brainstem level. The preparation of movements can be tested with a StartReact paradigm; StartReact refers to the accelerated release of a prepared motor program when a startling acoustic stimulus (SAS) is presented simultaneously with the imperative stimulus [6, 27]. This phenomenon is explained by the SAS directly releasing a pre-programmed motor response at the level of the reticular formation [27]. Using this paradigm, McCombe Waller and colleagues indeed found evidence for impaired motor preparation of both anticipatory postural adjustments and arm movements, during standing reaches after stroke [21]. In contrast, preserved motor preparation in people with stroke has been demonstrated for ballistic upper extremity movements in several other studies [13, 14, 20].

The aim of this study was twofold. First, we aimed to verify whether the intact StartReact effects that have previously been demonstrated for ballistic upper extremity movements also pertain to a single-joint lower extremity movement (i.e. ankle dorsiflexion) in people with stroke. Second, we aimed to investigate the StartReact effect on APRs following a translational perturbation of the support surface that induced a backward body movement. We studied the effects of a SAS on the onset latencies of tibialis anterior (both tasks) and rectus femoris responses (only applicable to the perturbations) as the primary outcomes. Our hypothesis was that ankle dorsiflexion and APR movement preparation in people with stroke would be unaffected compared to control subjects. In the people with stroke, an elimination of onset delays and normalization of inter- and intra-limb coordination (during APRs only) when pairing the task with a SAS would support this hypothesis.

## Materials and methods

### Participants

In this study, 12 people in the chronic phase after unilateral supratentorial stroke and 12 healthy controls participated. Stroke survivors (> 6 months after stroke) were included if their stroke had resulted in a contralateral hemiparesis. Furthermore, the participants had to be able to stand bare feet and had to have (corrected to) normal vision and hearing. Exclusion criteria were any impairments (unrelated to hemiparesis) or use of medication that could influence balance capacity or reaction times (e.g. neuroleptics and benzodiazepines), and severe cognitive impairment (Mini-Mental State Examination score < 24). The study was approved by the medical ethics committee of the region Arnhem-Nijmegen and was conducted in accordance with the Declaration of Helsinki. All participants gave written informed consent prior to the experiment.

### Clinical assessments

In people with stroke, motor selectivity was determined by the Fugl-Meyer assessment for lower extremities (FM-L; range 0–34) with higher scores indicating better motor recovery [10]. In addition, muscle strength was assessed using the lower limb items of the Motricity Index (range 0–33 for each item) with higher scores indicating more muscle strength [9]. Moreover, muscle tone was rated for the paretic lower limb using the Modified Ashworth Scale (MAS; range 0–4 for each item) with higher scores implying more severe hypertonia [2]. Balance capacity was assessed with the Berg Balance Scale (BBS; range 0–56) with higher scores indicating better static and dynamic balance abilities [1].

### Experimental protocol

Each participant performed two tasks: the first was a simple voluntary reaction time task involving a ballistic ankle dorsiflexion movement. The second task involved responding to sudden external balance perturbations. Each task consisted of 16 experimental trials, preceded by 4 practice trials.

### Ankle dorsiflexion task

Reaction times of ankle dorsiflexion movements were evaluated for both legs. People with stroke started with their unaffected leg, whereas controls started with their dominant leg (identified by the question ‘with which foot would you kick a soccer ball?’). Participants sat on a height-adjustable chair with knees and ankles in a 90° angle in front of two arrays of light-emitting diodes (LEDs; 11 × 8 cm, 3 cm apart). The left array served as a warning sign and, after a variable interval (1–3.5 s), illumination of the right LED array was the ‘go’ signal to which the participant had to perform an ankle dorsiflexion movement as fast as possible. In 25% of the trials, a Startling Acoustic Stimulus (SAS) was released through binaural headphones simultaneously with the go signal. A custom-made noise generator was used to produce the SAS (50 ms of 1500 Hz white noise at 120 dB Sound Pressure Level). SAS were randomly released across trials, but not on the first trial and never twice in succession.

### Balance perturbations

This task involved recovering from backward balance perturbations with a feet-in-place strategy (i.e. without taking a step or grasping handrails for support). Backward body perturbations were delivered on the Radboud Falls Simulator (240 × 174 cm; BAAT, Enschede, The Netherlands) [23] by means of an anterior translation of a moveable platform. The perturbation waveform comprised an acceleration phase of 300 ms at 0.500 m/s^2^, followed by a constant velocity phase of 500 ms and a deceleration phase of 300 ms. A variable delay of ~ 7 to 10 s was added to the time between two consecutive trials. All participants stood with their feet 4.5 cm apart and wore a safety harness attached to the ceiling to prevent falling. Furthermore, people with stroke wore a soft ankle brace (ASO, Medical Specialities, Wadesboro, NC, USA) on the paretic side to prevent possible ankle sprains due to the imposed perturbation. Note that the brace restricted inversion and eversion movements, while ankle plantar and dorsiflexion was not limited within the range of motion needed for the feet-in-place strategies. Here too, in 25% of the trials, a SAS was released simultaneously with the onset of the platform movement.

### Data sampling and analysis

Surface electromyography (EMG) was recorded bilaterally from the tibialis anterior (TA), rectus femoris (RF), and sternocleidomastoid muscles (SCM) (ZeroWire, Aurion, Italy) according to Seniam guidelines [11] and sampled at 2000 Hz. All EMG signals were bandpass filtered at 20–450 Hz (zero lag, second-order Butterworth filter), full-wave rectified and low-pas filtered at 30 Hz (zero lag, second-order Butterworth filter). An accelerometer was placed on the dorsum of each foot to detect foot movement during ankle dorsiflexion movements to ensure that the SAS did not only result in shortened EMG onsets, but also in shortened movement onsets [25]. Acceleration trajectories were sampled at 2000 Hz and then low-pas filtered at 30 Hz (zero lag, second-order Butterworth filter). Reflective markers were placed on the spinous process of the seventh cervical vertebra (C7) and on top of the moveable platform during balance perturbations. The marker trajectories were recorded at 100 Hz by an 8-camera 3D-motion analysis system (Vicon Motion Systems, United Kingdom) and then low-pass filtered at 10 Hz (zero lag, second-order Butterworth filter).

EMG and accelerometer signals were ensemble averaged for non-SAS trials and SAS trials separately. A semi-automatic algorithm determined muscle and movement onsets as the first instant where the ensemble-averaged signal exceeded two times the SD of the mean background EMG activity (as measured during the 500 ms time interval prior to the ‘go’ signal or the start of the platform movement) [24]. All onsets were visually verified and corrected if necessary. In controls, onset latencies were averaged across legs. In addition, maximum posterior body excursion following a balance perturbation was determined from the C7 marker trajectory (after subtracting the trajectory of the platform marker). For each trial with a SAS, we also determined whether a startle reflex occurred in SCM. A startle reflex was defined as short latency response in any of the SCM muscles starting within 130 ms following the SAS. MATLAB 2014b (MathWorks, Natick, MA, USA) was used for all data operations.

### Statistical analysis

To test whether, in either task, muscle onset latencies with and without SAS differed between the non-paretic legs of stroke participants and controls, we used a repeated measures ANOVA with group (non-paretic leg vs. controls) as between-subjects factor and SAS as within-subject factor. To determine whether differences existed between the paretic and the non-paretic legs of the stroke participants, we conducted a repeated measures ANOVA with leg and SAS as within-subject factors. To determine whether the administration of the SAS had an effect on intra- and inter-limb coordination during APRs, coefficients of determination (*R*^2^) were calculated from Pearson’s correlation coefficients between TA and RF onsets in the same leg (intra-limb coordination), and between bilateral onsets of TA and RF (inter-limb coordination) for trials with and without SAS separately. Furthermore, to determine whether C7 excursions with and without SAS differed between stroke participants and controls, a repeated measures ANOVA was used with group (stroke vs. controls) as the between-subjects factor and SAS as within-subject factor. As a secondary analysis, we used a paired-samples *t*-test to identify potential differences in TA onset latencies between SAS trials without (SCM−) and with a startle reflex in the sternocleidomastoid muscle (SCM+), for individuals who presented both. This secondary analysis was performed because there is an ongoing debate on whether the StartReact effect critically depends on the occurrence of a startle reflex. As such, this analysis was used to determine the potential impact of our decision of analyzing all SAS trials (as opposed to only including SCM+ trials) on our primary results and conclusions. The alpha level was set at 0.05. All statistical analyses were performed in IBM SPSS 22 (SPSS, Inc., Chicago, IL, USA).

## Results

Participants’ demographics and clinical characteristics are presented in Table 1.

**Table 1:** Participants’ characteristics

	Stroke (♂:7/♀:5)	Control (♂:7/♀:5)	*t* test *p* value
Age (years)	62 (9.6)	66 (8.8)	0.272
Height (m)	1.70 (0.1)	1.70 (0.1)	0.933
Weight (kg)	78.3 (9.9)	73.7 (13.5)	0.346
BMI	27 (2.8)	25.4 (3.4)	0.192
Time post stroke (years)	7 (4.9)		
Paretic side (left/right)	6/6		
Type of stroke (ischemic/hemorrhagic)	10/2		
Berg balance scale (0–56)	52 (5.8)		
FM-L Score (0–34)	26 (4.9)		
Motricity Index (0–33)			
Ankle dorsiflexion	22.7 (7.4)		
Knee extension	24.0 (6.4)		
Hip flexion	26.7 (5.2)		
MAS (0–4)			
Knee extension	0 [0–1+]		
Knee flexion	0 [0–2]		
Ankle dorsiflexion	0 [0–2]		
Ankle plantarflexion	0 [0–0]		

Data are presented as ‘mean (standard deviation)’, ‘median [range]’, or number of participants

*BMI* body mass index, *FM-L* Fugl-Meyer assessment for lower extremity, *MAS* modified ashworth scale

### Ankle dorsiflexion task

All participants were able to perform the ankle dorsiflexion movements. Although in some people with stroke, the movement was small on the paretic side, movement onsets were detectable from the accelerometer signal.

**Fig. 1:**
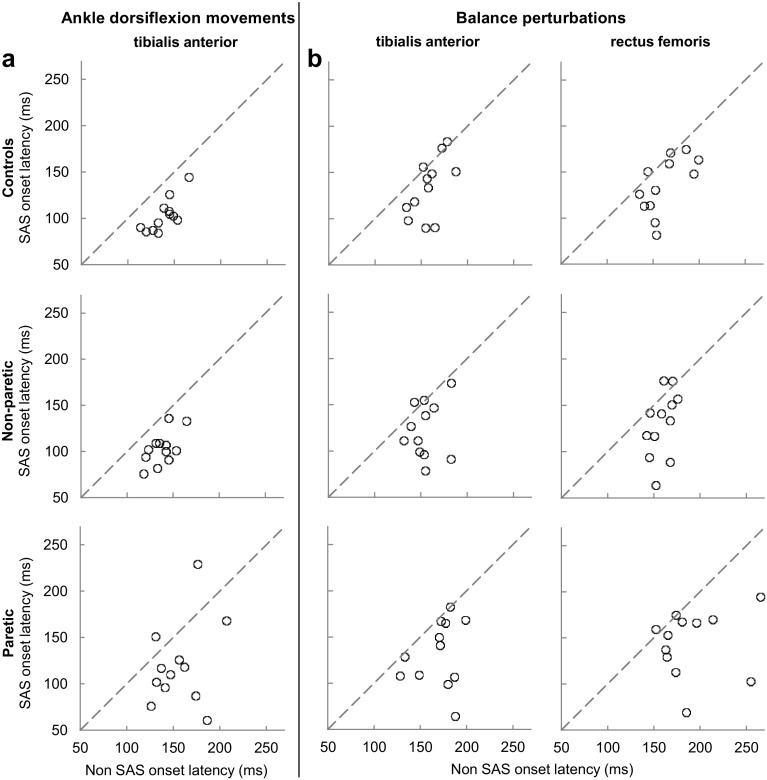
Relation between onset latencies without SAS and with SAS for tibialis anterior during ankle dorsiflexion movements (left column) and during the balance perturbation task (middle column) and rectus femoris (right column) during balance perturbations. Relations are displayed for controls (upper graphs), the non-paretic leg (middle graphs) and the paretic leg (lower graphs) of participants with stroke. Dots (circle) below the dashed lines indicate an acceleration of the onset latency by the SAS for a particular subject

Figure 1a shows the individual TA onset latencies of all participants for SAS and non-SAS trials. All data points below the dashed line indicate that TA onsets were generally accelerated by the SAS. From this figure it is evident that both in controls and in the non-paretic leg of the people with stroke, without exception, TA onsets were faster with than without SAS. In the paretic leg, however, two participants showed delayed TA onsets when the SAS was administered.

### Non-paretic leg vs. controls

**Fig. 2:**
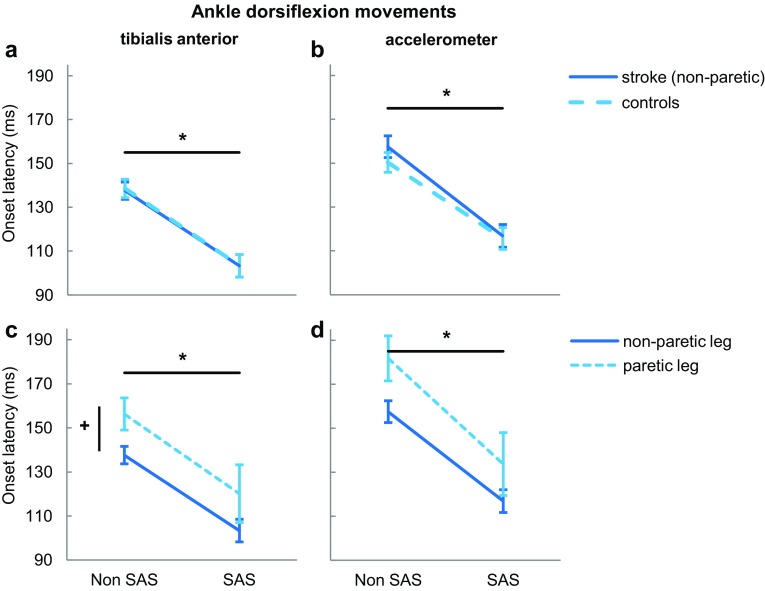
Onset latencies ± standard error of the mean of ankle dorsiflexion movements, without and with SAS. Onsets of tibialis anterior are shown in the left graphs. The right graphs show movement onsets measured by an accelerometer on the dorsal area of the foot. *Indicates a significant within-subjects effect (*p* < 0.001). ^+^Indicates a significant within-subjects effect (*p* < 0.05)

TA onsets of the non-paretic legs were not different compared to those of control legs (Group: *F*_1,22_ = 0.008, *p* = 0.932; Fig. 2a). TA onset latencies were shorter for SAS trials than for non-SAS trials in both the non-paretic leg of people with stroke (103 ± 17.8 vs. 138 ± 13.7 ms) and in controls (103 ± 17.7 vs. 139 ± 14.6 ms; SAS: *F*_1,22_ = 3,255, *p* < 0.001), which effect was not different between groups (SAS × Group: *F*_1,22_ = 0.034, *p* = 0.856). Similar results were found for foot movements as detected by the accelerometer (stroke: 117 ± 17.9 vs. 158 ± 17.2 ms; control: 116 ± 17.2 vs. 150 ± 15.6 ms; Group: *F*_1,22_ = 0.425, *p* = 0.521; SAS: *F*_1,22_ = 193.879, *p* < 0.001; SAS × Group: *F*_1,22_ = 1.230, *p* = 0.279; Fig. 2b).

### Paretic vs. non-paretic leg

As shown in Fig. 2c, TA onsets in the paretic leg were on average 18 ms later than in the non-paretic leg (Leg: *F*_1,11_ = 4.900, *p* = 0.049). The SAS shortened TA onsets during the ankle dorsiflexion movements to a similar extent in the paretic (120 ± 45.4 vs. 156 ± 25.3 ms) and non-paretic leg (SAS: *F*_1,11_ = 24.676, *p* < 0.001; SAS × Leg: *F*_1,11_ = 0.022, *p* = 0.886). Similar results were found for foot movements as measured by the accelerometer (Leg: *F*_1,11_ = 4.575, *p* = 0.056; SAS: *F*_1,11_ = 37.182, *p* < 0.001; SAS × Leg: *F*_1,11_ = 0.504, *p* = 0.493; Fig. 2d).

### Balance perturbations

All participants were able to sustain the backward balance perturbations without taking a step.

Figure 1b shows the individual TA and RF onset latencies of all participants for SAS and non-SAS trials. Data points below the dashed line indicate that onsets latencies were shortened by the SAS. In general, TA and RF onsets were faster with than without SAS in either group, yet a relatively large heterogeneity was present in the paretic leg of the people with stroke.

### Non-paretic leg vs. controls

**Fig. 3:**
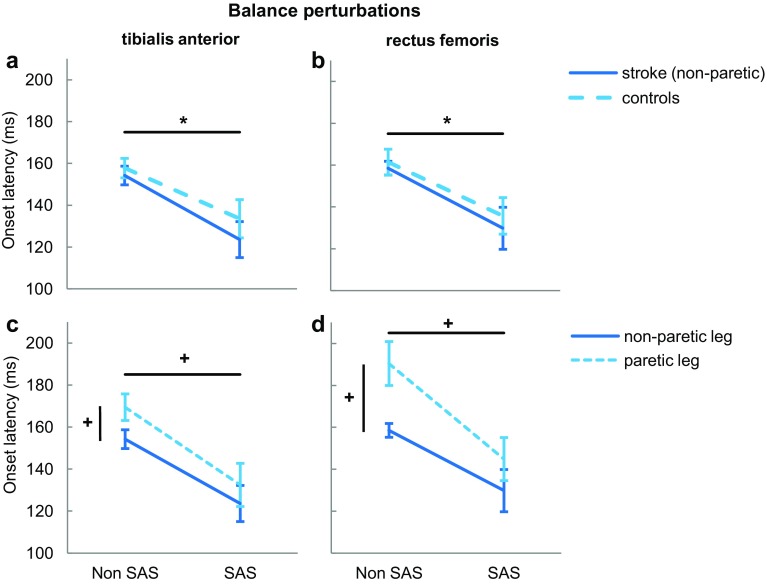
Onset latencies ± standard error of the mean of tibialis anterior (left graphs) and rectus femoris (right graphs) during balance perturbations, without and with SAS. *Indicates a significant within-subjects effect (*p* < 0.001). ^+^Indicates a significant within-subjects effect (*p* < 0.05)

Figure 3 shows that the onset latencies in the non-paretic leg were not significantly different from those in controls, which was true for both TA and RF (Group: TA: F_1,22_ = 0.695, *p* = 0.413; RF: *F*_1,22_ = 0.255, *p* = 0.619; Fig. 3a, b). In non-paretic and control legs, onset latencies were accelerated by a SAS compared to non-SAS trials in TA (stroke: − 31; control: − 24 ms; SAS: *F*_1,22_ = 22.177, *p* < 0.001) and RF (stroke: − 29 ms; control: − 26 ms; SAS: *F*_1,22_ = 22.958, *p* < 0.001). The SAS-induced acceleration of TA and RF onset latencies was similar for non-paretic and control legs (SAS × Group: TA: *F*_1,22_ = 0.301, *p* = 0.588; RF: *F*_1,22_ = 0.074, *p* = 0.789).

### Paretic vs. non-paretic leg

Paretic TA and RF onset latencies were significantly slower (12 and 23 ms, respectively) compared to the non-paretic leg (Leg: TA: *F*_1,11_ = 5.005, *p* = 0.047; RF: *F*_1,11_ = 8.416, *p* = 0.014; Fig. 3c, d). The SAS accelerated the onsets in both the paretic (TA: − 37 ms; RF: − 45 ms) and non-paretic leg (TA: *F*_1,11_ = 12.261, *p* = 0.005; RF: *F*_1,11_ = 12.870, *p* = 0.004), yet again, no interaction effect was found (SAS × Leg: TA: *F*_1,11_ = 1.358, *p* = 0.269; RF: *F*_1,11_ = 2.546, *p* = 0.139).

### C7 excursions

**Fig. 4:**
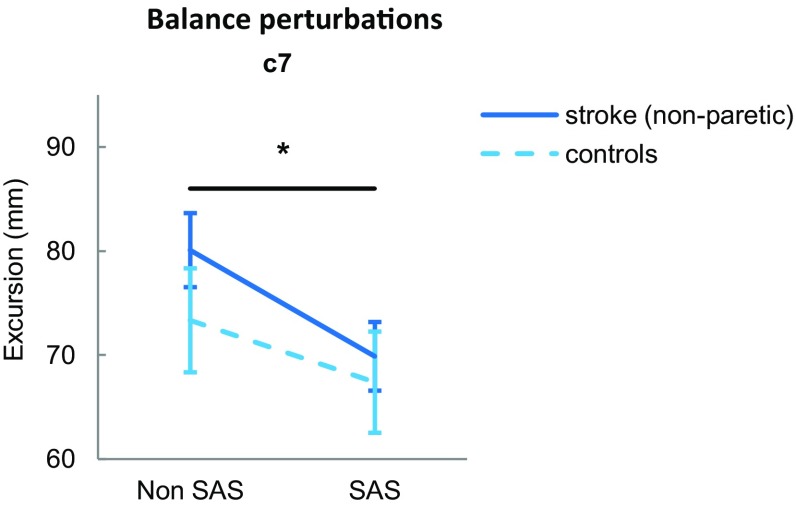
Amount of postural sway after postural balance perturbations measured by the excursion of C7 in the backward direction. ^*^Indicates a significant within-subjects effect (*p* < 0.001)

C7 excursions were not different between people with stroke and controls (Group: *F*_1,22_ = 0.662, *p* = 0.425; SAS × Group: *F*_1,22_ = 54.285, *p* = 0.296; Fig. 4). The SAS similarly reduced the C7 excursions for people with stroke (70 ± 11.4 vs. 80 ± 12.3 mm) and controls (67 ± 16.9 vs. 73 ± 17.3 mm; SAS: *F*_1,22_ = 16.521, *p* = 0.001).

### Intra- and inter-limb coordination

**Fig. 5:**
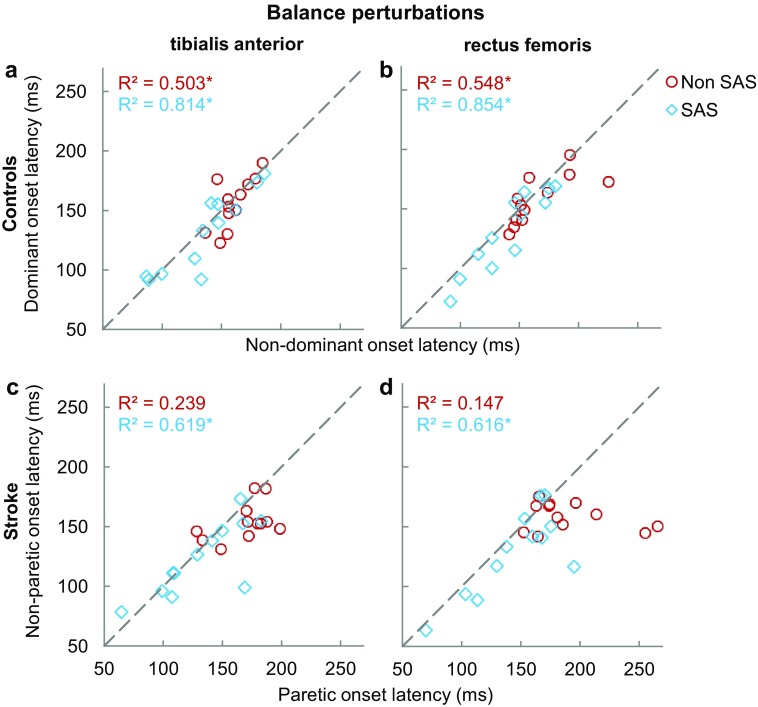
Inter-limb coordination: relation between onset latencies of both legs without SAS (red circle) and with SAS (blue diamond), with controls presented in the upper graphs and participants with stroke in the lower graphs. Onset latencies during the balance perturbations are displayed for tibialis anterior in panel a and c and for rectus femoris in panel b and d. ^*^Indicates significant correlation (*p* < 0.05)

Figure 5 shows how following a backward balance perturbation, TA and RF onset latencies were associated between the legs (inter-limb coordination). For the controls, the individual data points tightly clustered around the dashed line, indicating similarity in TA and RF onsets between the legs, whereas for the stroke participants greater deviations from the dashed line were observed. Following balance perturbations, the statistical analyses indeed yielded significant and strong between-leg associations in the healthy controls, which relationships became even stronger when the SAS was administered, both in TA (non-SAS: *R*^2^ = 0.503, *p* = 0.010; SAS: *R*^2^ = 0.814, *p* < 0.001; Fig. 5a) and in RF (non-SAS: *R*^2^ = 0.548, *p* = 0.006; SAS: *R*^2^ = 0.854, *p* < 0.001; Fig. 5b). In the stroke participants, these relationships between the paretic and non-paretic leg in the non-SAS condition were only weak to moderate (TA: *R*^2^ = 0.239, *p* = 0.106; RF: *R*^2^ = 0.147, *p* = 0.337; Fig. 5c, d) and did not reach significance. In the SAS condition, however, between-leg associations in TA and RF became substantially stronger (TA: *R*^2^ = 0.619, *p* = 0.002; RF: *R*^2^ = 0.616, *p* = 0.003).

**Fig. 6:**
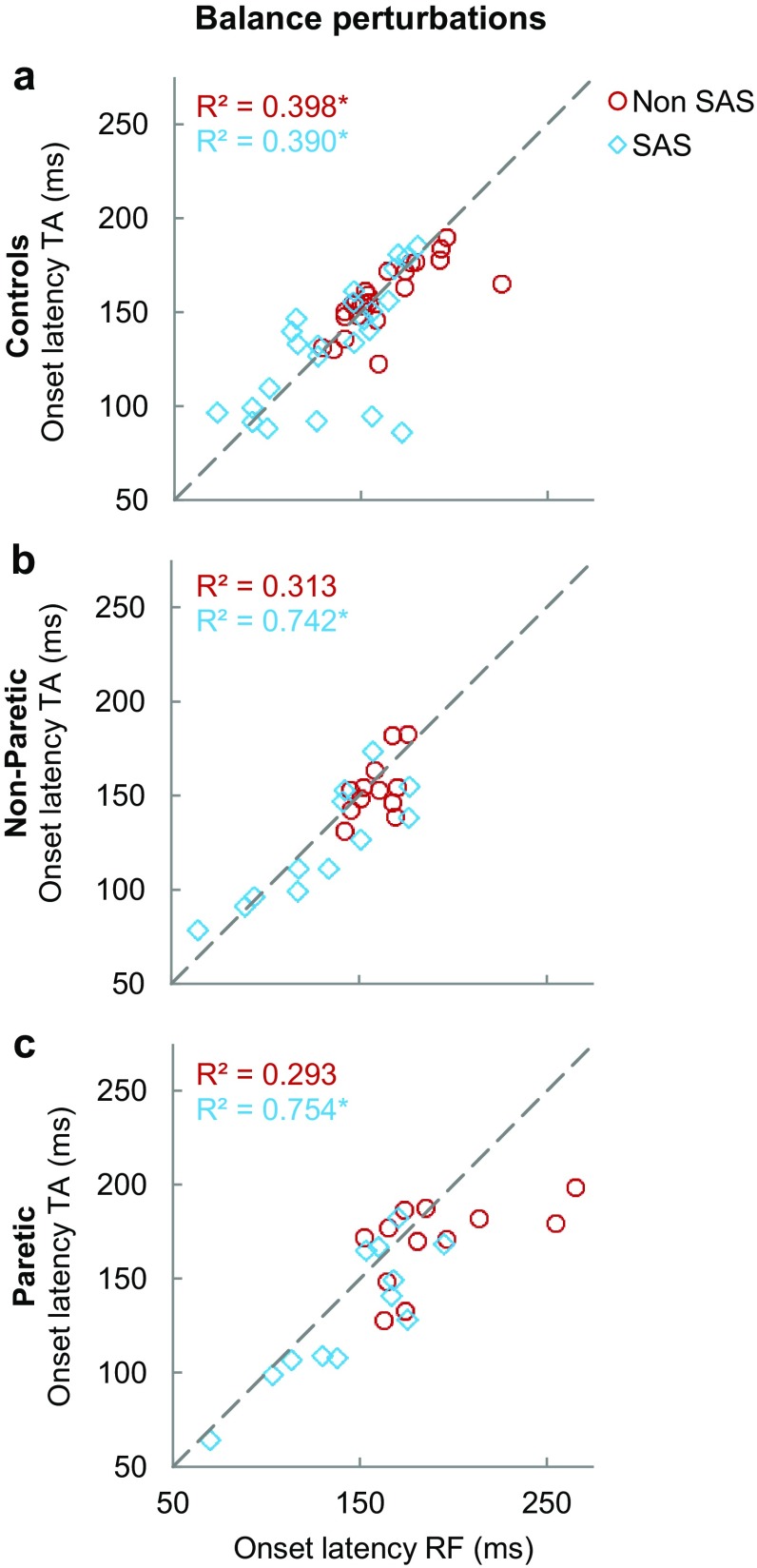
Intra-limb coordination: relation between onset latencies of tibialis anterior (TA) and rectus femoris (RF) within the same leg for perturbations without SAS (red circle) and with SAS (blue diamond). Both legs of controls are presented in the upper graph (**a**), whereas the non-paretic leg (**b**) and paretic leg (**c**) of the stroke participants are depicted in the lower graphs. ^*^Indicates significant correlation (*p* < 0.05)

Figure 6 shows the intra-limb associations between TA and RF onsets following balance perturbations. In the controls, TA and RF onset latencies showed moderately strong associations both without (*R*^2^ = 0.398, *p* < 0.001) and with SAS (*R*^2^ = 0. 390, *p* = 0.001; Fig. 6a). In the stroke participants, these associations were slightly weaker and non-significant in either leg in the condition without SAS (non-paretic: *R*^2^ = 0.313, *p* = 0.059; paretic: *R*^2^ = 0.293, *p* = 0.069; Fig. 6b, c). In the SAS condition, however, strong and highly significant intra-limb associations were found between TA and RF onsets for both the non-paretic and paretic leg (non-paretic: *R*^2^ = 0.742, *p* < 0.001; paretic: *R*^2^ = 0.754, *p* < 0.001).

### Startle reflexes in SCM

The rates of occurrence of a startle reflex in SCM were not different in stroke participants compared to controls. This was true for both the ankle dorsiflexion task (67% SCM + trials in the stroke group vs.73% in controls, *t*_22_ = − 0.485, *p* = 0.633) and the balance perturbations (46% in people with stroke vs. 54% in controls, *t*_22_ = − 0.627, *p* = 0.537). To determine whether the occurrence of a startle reflex in SCM had a differential effect on the aforementioned SAS-induced accelerations, we compared TA onsets between SCM+ and SCM− trials. For ankle dorsiflexion movements, this analysis was restricted to 15 participants (eight people with stroke) who showed both SCM + and SCM− trials. Within these participants, TA onset latencies were not different for SCM + trials compared to SCM− trials (129 ± 26.7 vs. 128 ± 34.4 ms; *t*_14_ = 0.265, *p* = 0.795). Moreover, there was no difference between SCM+ and SCM− trials in TA onset (146 ± 28.4 vs. 148 ± 26.8 ms; *t*_16_ = 0.235, *p* = 0.817) in 17 participants (seven people with stroke) during balance perturbations.

## Discussion

The aim of the current study was to investigate whether administration of a SAS simultaneously with a backward standing balance perturbation would speed up automatic postural responses (APRs) in participants after stroke. We further investigated whether the SAS would improve inter- and intra-limb muscular coordination during APRs. As expected, the paretic leg of stroke participants showed delayed onset latencies of tibialis anterior (TA) and rectus femoris (RF) compared to the non-paretic leg and to controls. We found that a SAS accelerates bilateral onset latencies of APRs not only in healthy controls, but for the first time, we also demonstrated this StartReact effect on APRs in people with stroke. Moreover, after stroke, inter- and intra-limb muscular coordination was rather weak without the SAS, but substantially improved when the SAS was applied. In addition, the participants also performed voluntary ankle dorsiflexion movements in response to a visual cue with and without a SAS. The pronounced SAS-induced acceleration of ankle dorsiflexion movements at the paretic side shows that the StartReact effect is intact for single-joint lower extremity movements. These findings suggest some degree of preserved movement preparation of APRs and single-joint lower extremity movements in the chronic phase after stroke.

The finding that a SAS accelerates ankle dorsiflexion movements is in line with findings from a previous study of our group in people with retrograde degeneration of the corticospinal tract (hereditary spastic paraplegia, HSP) [25]. It also shows agreement with studies on ballistic upper extremity movements in people with stroke, which demonstrated intact StartReact effects (i.e. muscle onset latencies were significantly reduced with SAS) on the paretic side [13, 14, 20]. In the present experiment we not only studied the paretic, but also the non-paretic side. We found that reaction times of ankle dorsiflexion movements at the non-paretic side did not differ from those in healthy controls, neither with nor without a SAS. Hence, the present study shows that the StartReact effect applies to both paretic and non-paretic lower extremity movements in people with stroke, albeit with some minor residual delay (on average 17 ms) in the paretic compared to the non-paretic side.

Importantly, we here demonstrate that the SAS not only accelerates voluntary ballistic movements at the paretic side, but also speeds up bilateral automatic postural responses (i.e. TA and RF onsets after backward balance perturbations) in people with hemiparetic stroke. These results are very similar to previous studies in healthy young subjects [4, 26] and thereby provide further support that StartReact effect indeed applies to balance recovery responses after external perturbations. Furthermore, Nonnekes and colleagues demonstrated that a SAS not only yielded earlier muscle onset latencies, but also coincided with smaller body excursions following the perturbation. Correspondingly, we here found that in the people with stroke, the reduction in APR onset latencies was paralleled by a reduction in postural sway as well. Our findings of significant SAS-induced accelerations of whole-body movements in people with stroke, however, are in contrast to those reported by McCombe Waller and colleagues. In their study, in which participants also had to perform a whole-body movement (i.e. reaching while standing) with and without a SAS, the StartReact effect was present in healthy controls, but absent in people with stroke [21]. This inconsistency might be due to the poorer motor recovery in their study group compared to our group. For instance, the mean BBS score was lower in the study of McCombe Waller compared to the present study (41.5 vs. 52.0, out of a maximum of 56) indicating poorer balance capacity. It seems reasonable to assume that a more severe stroke leads to greater problems with motor preparation, because of potential affliction of premotor areas, the corticoreticular tract or both.

Besides accelerated APR onset latencies for individual muscles, we also found that the SAS substantially enhanced intra- and inter-limb muscle coordination. While in controls inter-limb onset latencies already demonstrated significant and moderately strong associations without SAS in both TA and RF, we did not observe such inter-limb coupling in the stroke participants. Administration of the SAS, however, resulted in a substantial strengthening of the associations between paretic and non-paretic TA and RF onset latencies. Furthermore, weak intra-limb coupling (*R*^2^ = 0.293–0.313) between paretic TA and RF was observed without SAS, whereas associations between the onsets of these muscles became very strong with SAS (*R*^2^ = 0.742–0.754). The weak paretic TA-RF coupling in the condition without SAS is in line with the results of a previous study on postural responses in people with stroke [17]. These authors demonstrated its clinical significance, as the defective TA-RF coupling in the paretic leg was associated with an increased risk of falling. The substantial strengthening of paretic intra-limb coupling with a SAS concurs with the observations from a study that used an elbow flexion task in people with stroke, where coupling of paretic agonist and antagonist muscles normalized with administration of a SAS [13]. These collective findings suggest that *preparation* of coordinated movements across multiple muscles is preserved after stroke, but that lower excitability of postural circuits may be hampering the *execution* of APRs. Similarly, a previous study in our group showed a reduction in the APR delay with higher intensity balance perturbations compared to lower intensity perturbations in people with stroke [8]. Yet, it remains to be established why people with stroke have such difficulty to execute these well-prepared movements in the absence of a SAS. Future work should disentangle which cortical areas may play a role in the downregulation of subcortical postural circuits after stroke, and whether this is potentially remediable through interventions.

Although the SAS greatly accelerated muscle onset latencies in our group of stroke participants, we should note that, even with SAS, the average values of the paretic side demonstrated a residual delay of ~ 10–20 ms compared to the non-paretic side and to the healthy control group. This observation is different from the complete normalization of ankle dorsiflexion reaction times in HSP patients [25], and of paretic hand extension [14] and elbow flexion and extension movements [13] in people with stroke. It must be mentioned, though, that the SAS-induced acceleration was highly variable across stroke participants (as can be seen in Fig. 1). Two stroke participants (both with FMA scores below the median) even showed a slight delay in their paretic ankle dorsiflexion reaction times when the SAS was applied, which is something that we have not previously observed in healthy individuals or in people with HSP performing the same experimental task. Without these two participants, the SAS-induced TA reaction times were comparable between the paretic leg of people with stroke (106 ms) and controls (103 ms). Similarly, Honeycutt et al. [14] also reported defective StartReact effects in the most affected stroke survivor in their study. Hence, defective motor preparation of the requested movement seems to have been present in a minority of the stroke participants in the present study and in the study of Honeycutt et al. These observations may be explained by more affected premotor areas and/or corticoreticular tract in some stroke patients. This suggestion, however, is only speculative at this point and calls for neuroimaging studies to identify which specific CNS lesions may underlie the lack of StartReact (and thus defective motor preparation) in some of the people with stroke.

Unlike some other authors [4, 12, 13], we included all SAS trials in our analyses, regardless of whether a startle reflex was observed in SCM. Some previous studies on upper limb movements have reported small differences in SAS-induced reaction times between SCM+ and SCM− trials and argued that a startle reflex appears to be conditional to the StartReact effect [6, 7, 14]. However, similar to the lack of differences between SCM+ and SCM− trials that we and others have previously reported for lower limb muscles [5, 24, 26] we here again failed to demonstrate any difference in the leg muscles onsets between trials with and without a startle reflex. We are, thus, confident that including SCM− trials in the analyses has not affected our study outcomes.

As the population of people with stroke is very heterogeneous, the results from our small and relatively well-recovered group of participants cannot be generalized to the stroke population at large. The sample size was also too small to establish a potential relationship between severity of residual post-stroke symptoms and defective StartReact. Another limitation was that MRI scans of lesion site and size were not available for the vast majority of our participants. For future studies, we therefore recommend including larger numbers of well-characterized people with stroke with a wide range of motor impairments. Another limitation was that the people with stroke wore an ankle brace on the paretic side to prevent possible ankle sprains due to the imposed balance perturbations. Although we cannot completely rule out the use of the ankle brace having influenced the results, this possibility is deemed highly unlikely because the brace did not limit the (small) ankle plantar and dorsiflexion movements observed during feet-in-place responses.

Nonetheless, the finding of intact motor preparation of both voluntary ankle movements and automatic postural responses in our group of stroke participants is of great interest, as it may indicate residual (subcortical) capacity for motor recovery after cortical stroke. We showed here that delayed APR onsets were likely due to lower excitability of postural circuits, which may be overcome by applying a SAS. It would be of interest to determine whether repeated perturbation sessions with SAS may also yield training effects in terms of faster APR onsets *without* SAS. As an alternative possibility for increasing the excitability of postural circuits, previous work from our group has shown that APR onsets may be accelerated with non-invasive brain stimulation [22]. Yet, it remains for further research to identify whether such novel approaches may be useful to exploit the suggested residual capacity for improving balance capacity after stroke.
